# Supplementary material for: Single particle cryo-EM reconstruction of 52 kDa streptavidin at 3.2 Angstrom resolution
Source: Nat Commun. 2019 Jun 3;10:2386. doi: 10.1038/s41467-019-10368-w (PMC6546690; doi:10.1038/s41467-019-10368-w)
Supplement: Supplementary file 1 — Supplementary Information [file 41467_2019_10368_MOESM1_ESM.pdf]

Supplementary Information

Single particle cryo-EM reconstruction of 52 kDa streptavidin at 3.2 Angstrom resolution

Fan et al.

Supplementary Figures

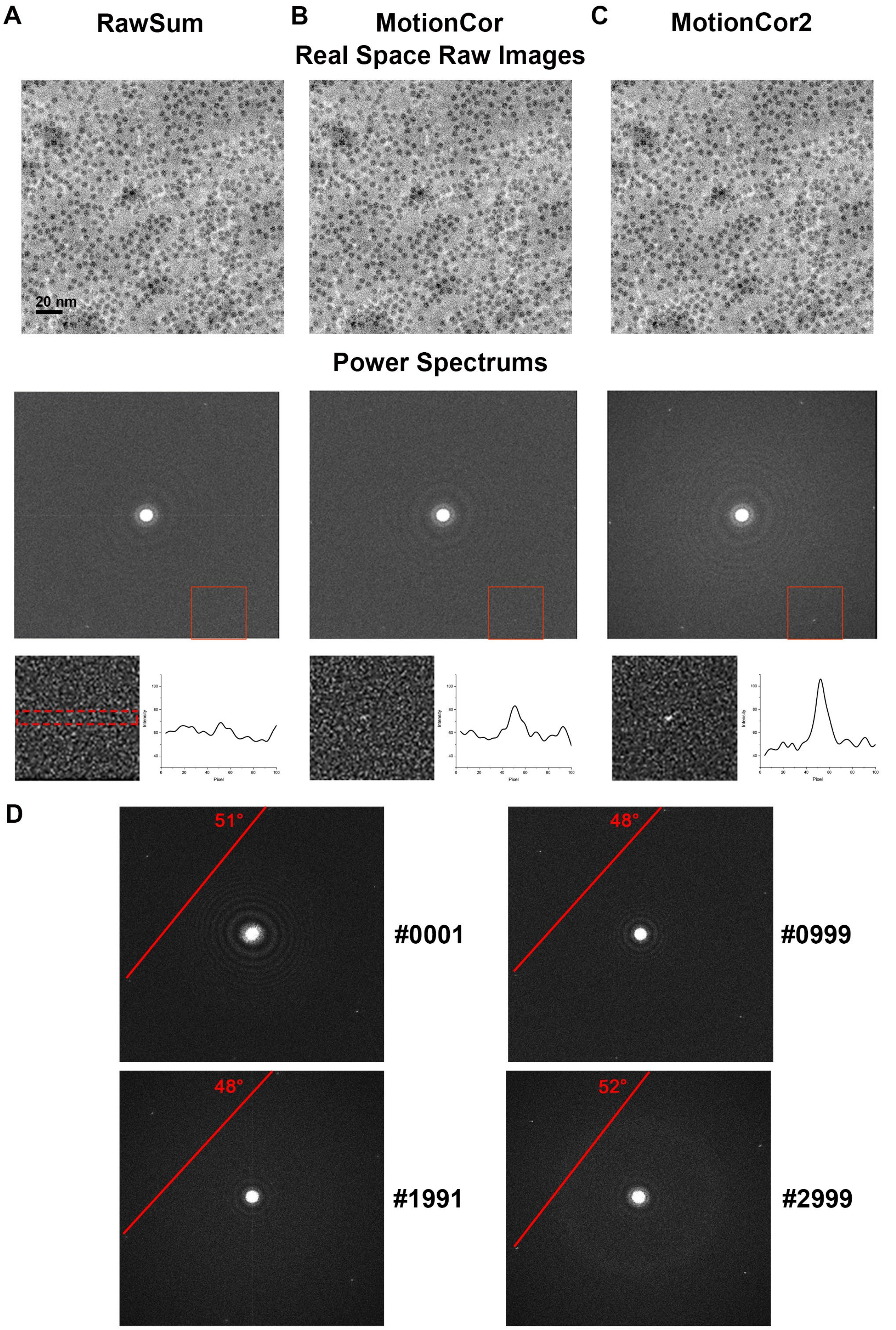

Supplementary Figure 1. Motion correction of movie stacks of SA specimen on graphene supporting film

(A-C) The summation of movie stacks from apo-SA dataset without motion correction, treated with MotionCor and with MotionCor2, respectively. In the top row are the summed images after correction. In the middle row are the corresponding power spectra calculated from the summed images. The reflections at 2.13 Angstrom resolution, reflecting the hexagonal lattice of graphene, are boxed in red. In the bottom row are the zoom-in of red squared region showing the reflection spot more clearly and the corresponding integrated intensity profile of each reflection within the red rectangular area as marked in the most left panel. (D) The Fourier transform of various areas (represented with different micrograph number) on the same specimen grid of biotin-SA. The direction of diffraction pattern was labeled in red. The differences in angles indicated a slight long range deformation of the graphene film.

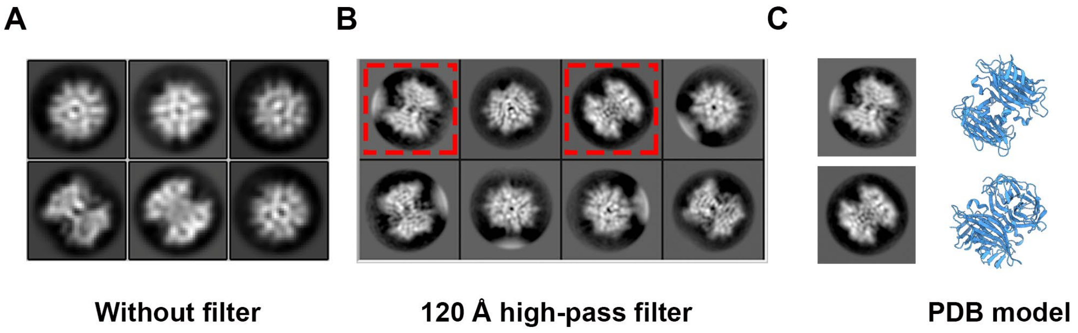

Supplementary Figure 2. High pass filtering for data processing
(A) Representative 2D class averages of SA particles without high-pass filtering. (B) Representative 2D class averages of SA particles with high-pass filtering at 120 Å. (C) Selected 2D class averages as boxed in (B) in comparison with the similar views of atomic model of SA (PDB 1MEP).

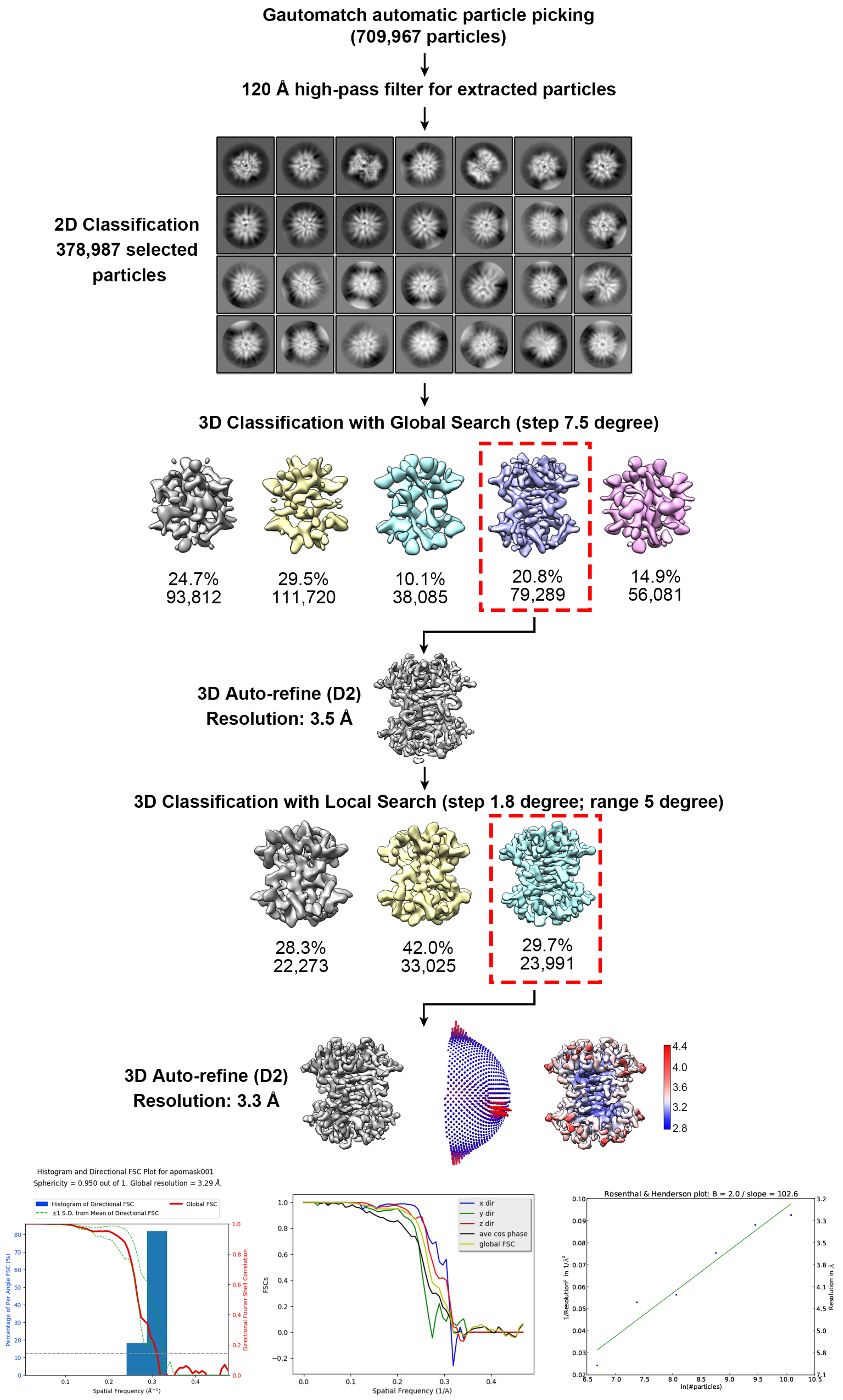

apo-SA data processing

Supplementary Figure 3. Single particle image processing flow-chart of apo-SA dataset.

The results from different image processing steps were presented, including 2D classification, 3D classification and final 3D auto-refine. Local resolution map, Euler angle distribution, directional FSC profile and B-factor plots were also presented.

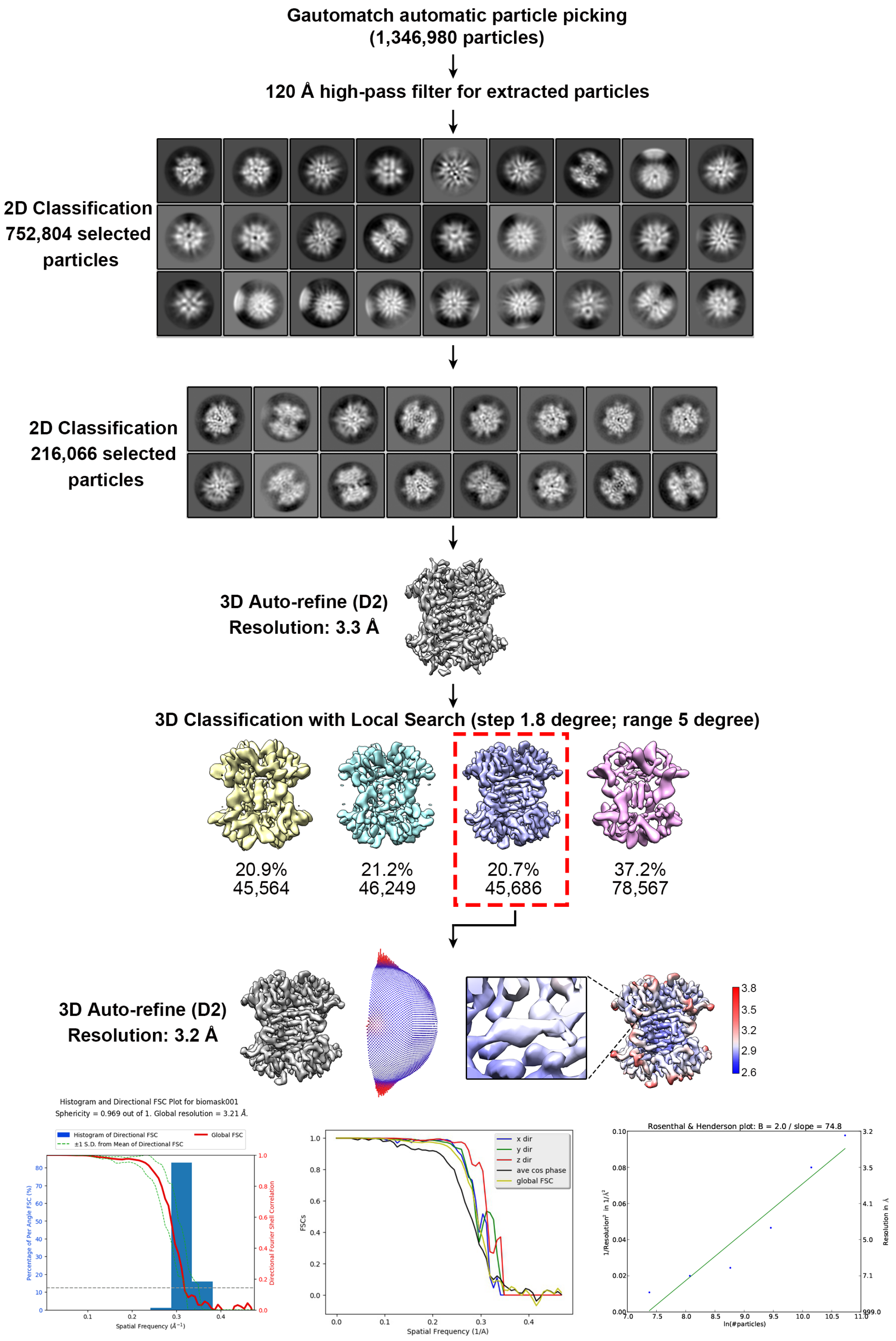

biotin-SA data processing

Supplementary Figure 4. Single particle image processing flow-chart of biotin-SA dataset.

The results from different image processing steps were presented, including 2D classification, 3D classification and final 3D auto-refine. Local resolution map, Euler angle distribution, directional FSC profile and B-factor plots were also presented.

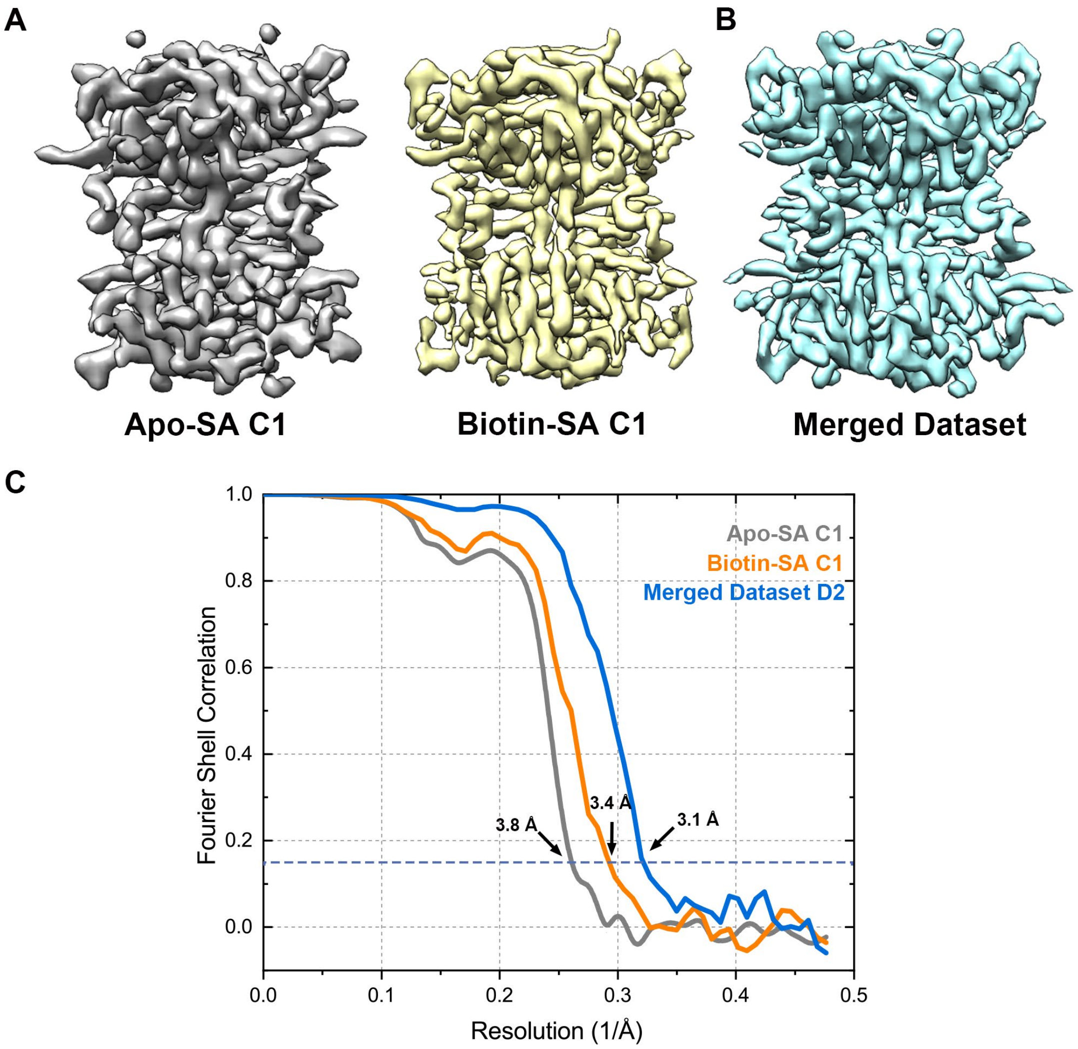

Supplementary Figure 5. Reconstructions of the SA datasets with different symmetry imposition

(A) The C1 symmetry reconstructions of apo-state SA (3.8 Å resolution) and biotin-bound SA (3.4 Å resolution), respectively. (B) The reconstruction of merged-dataset (apo-SA + biotin-SA) with D2 symmetry imposition (3.1 Å resolution). (C) The FSC curves of the three reconstructions with gold standard criteria.

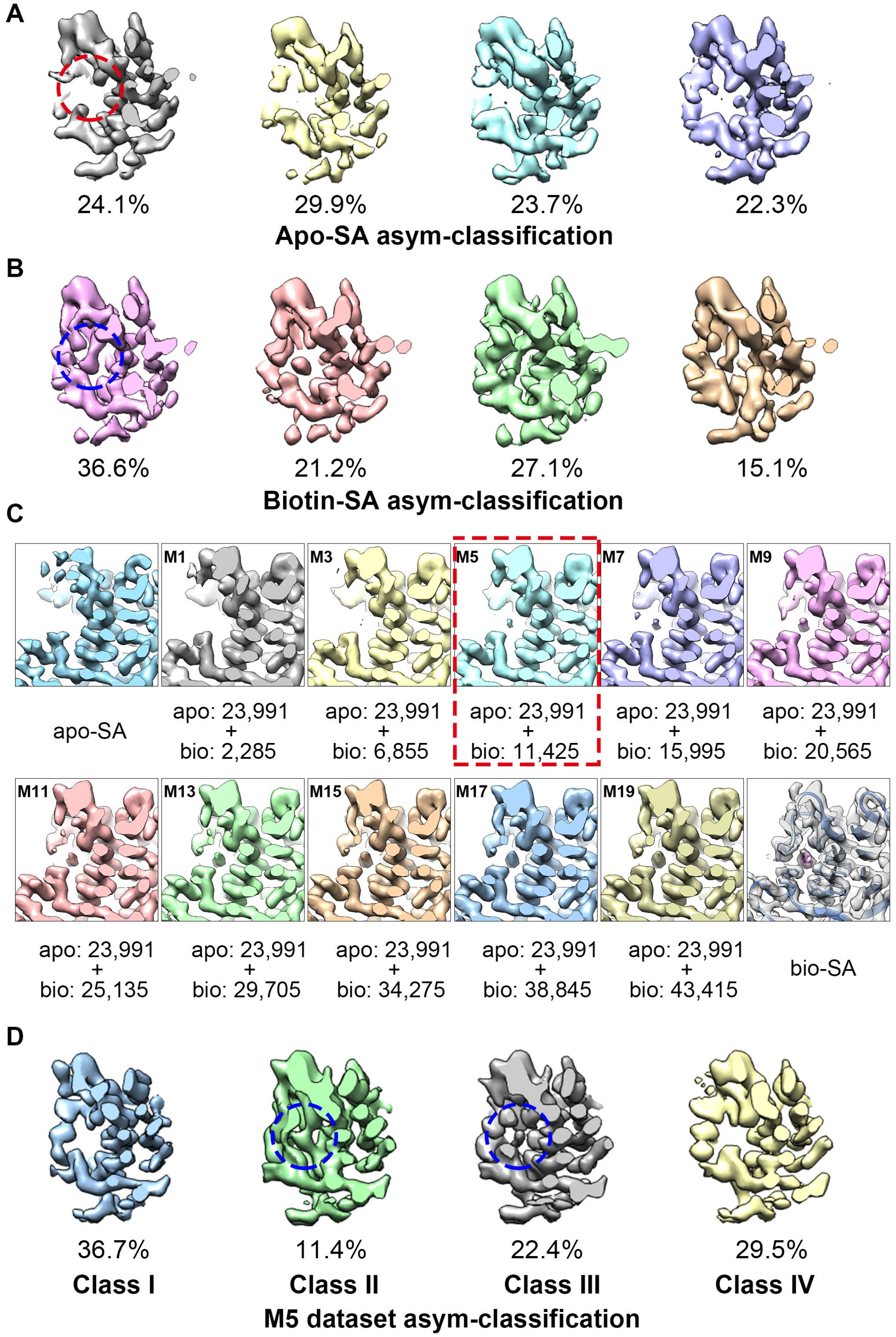

Supplementary Figure 6. Asymmetric classification of homogenous dataset of SA

(A) Asymmetric 3D classification of the apo-SA dataset demonstrating empty biotin-binding pockets (red circle) in all four classes. (B) Asymmetric 3D

classification of the biotin-SA dataset demonstrating occupied biotin-binding pockets (blue circle) in all four classes. (C) Biotin-SA dataset was randomly split into 20 subsets. 20 mixed datasets were generated by mixing apo-SA dataset with different numbers of biotin-SA subsets. The apo-SA dataset was mixed with 1 biotin-SA subset in M1 and with 3 biotin-SA subsets in M3, and so on. Ten representative reconstructions from mixed-datasets were presented as labelled, demonstrating gradually increased density of loop 46-51 and biotin molecule from the M5 mixed dataset. (D) Asymmetric 3D classification of the M5 mixed dataset in (C). Class I and IV show empty biotin-binding pockets. Class II and IIII show occupied biotin-binding pockets (blue circle). Source data are provided as a Source Data file.

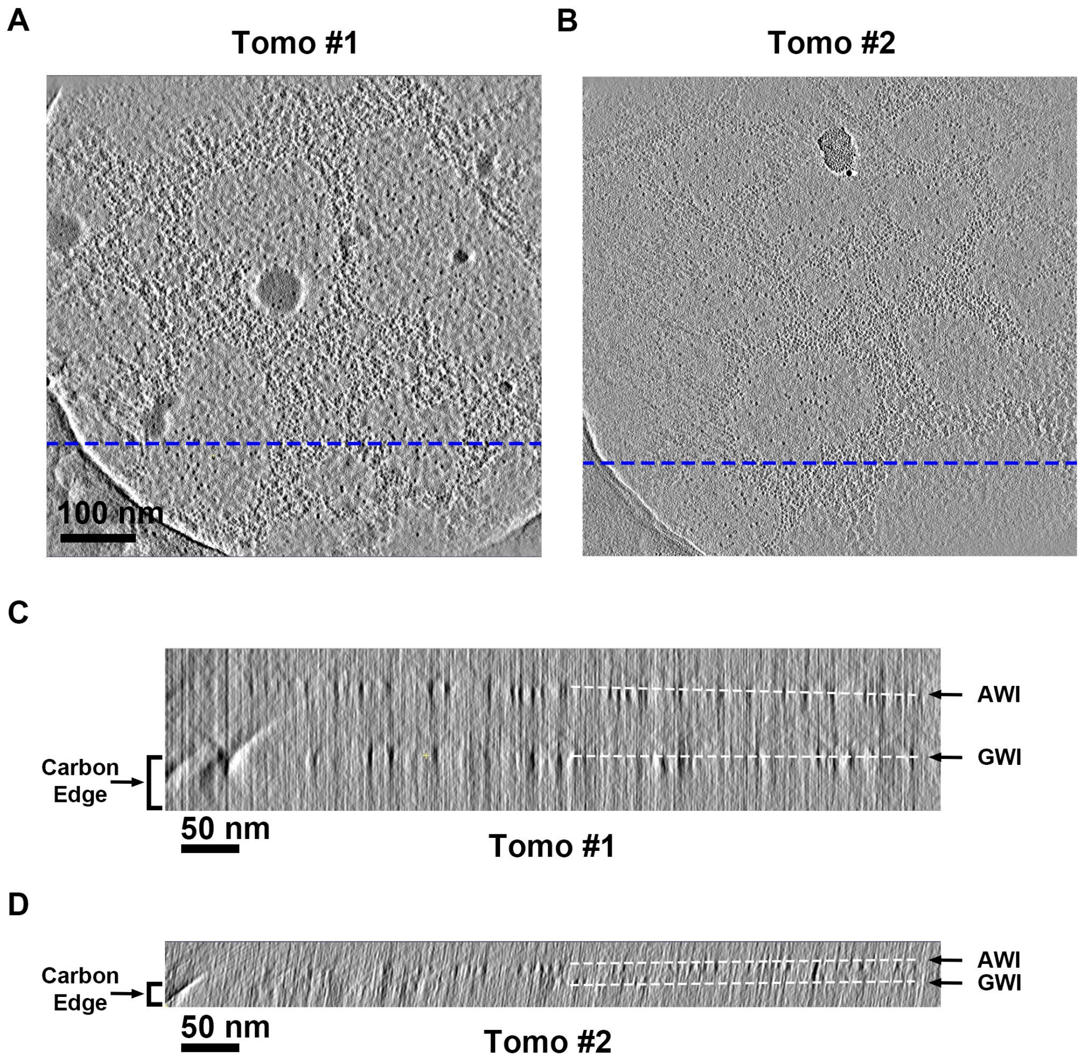

Supplementary Figure 7. Cross-sections from electron tomographic reconstructions

(A) and (B) are the X-Y cross-sections of graphene-water interface from two electron tomographic reconstructions. (C) and (D) are the X-Z cross-sections from the blue dash lines plane in the tomographic reconstructions of (A) and (B), respectively. White dash lines in (C) and (D) indicate the air-water interface (AWI) or graphene-water interface (GWI). The location of carbon edge of the holes are indicated in (C) and (D).

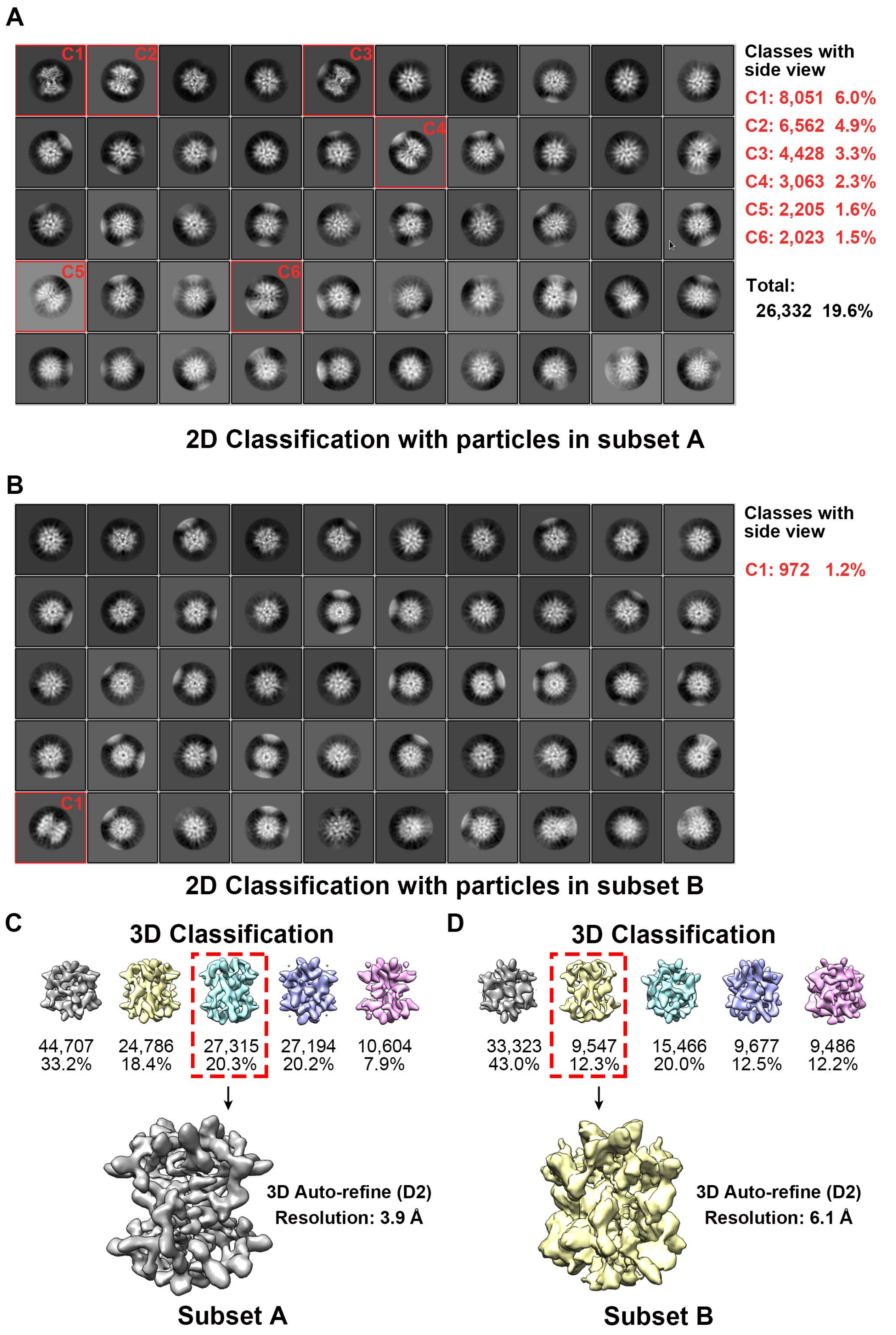

Supplementary Figure 8. Processing of apo-SA particles in two distinct subsets.

(A) 2D class averages of the Subset A particles. 6 classes (labeled as C1-C6 in red) demonstrate the butterfly-like side views. Particle number distribution in

the 6 classes is listed on the right. In total, 26,332 of 134,606 particles (19.6%) are in side-view. (B) 2D class averages of the Subset B particles. Only 1 class (C1, 972 particles, 1.2%) demonstrates the side view. 3D classification and subsequent 3D refinement were performed using particles in Subset A (C) or Subset B (D) individually. 27,315 selected particles in Subset A contributed to a 3.9 Å reconstruction with correct structural features (C). 9,547 selected particles in Subset B failed to give a reconstruction with correct structural feature due to the preferential orientation despite a reported 6.1 Å resolution (D). Source data are provided as a Source Data file.

After 3D Classification 44,326 particles for separate 3D-Refinement

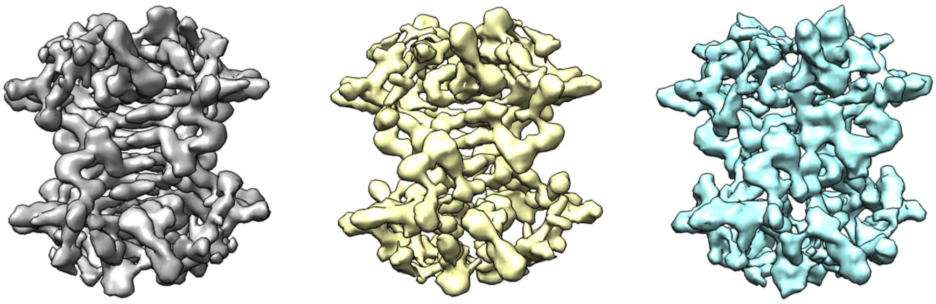

Subset A
35,676 particles
(3.5 Å)

Subset A
random split
8,650 particles
(4.0 Å)

Subset B
8,650 particles
(4.2 Å)

Supplementary Figure 9. Reconstructions of pre-selected apo-SA particles in the Subset A and Subset B.

Subset A particles and Subset B particles in the first round of 3D classification of the whole apo-SA dataset were used for 3D reconstruction. The left is the reconstruction from all the particles in Subset A and falling in the good class after the 3D classification. The right is the reconstruction from all the particles in Subset B and falling in the good class after the 3D classification. The middle is the reconstruction from a random split of particles from Subset A with the same number of particles for the right map. Their corresponding number of particles and resolution are labeled. All reconstruction were performed from local search using the angular information from the best reconstruction.

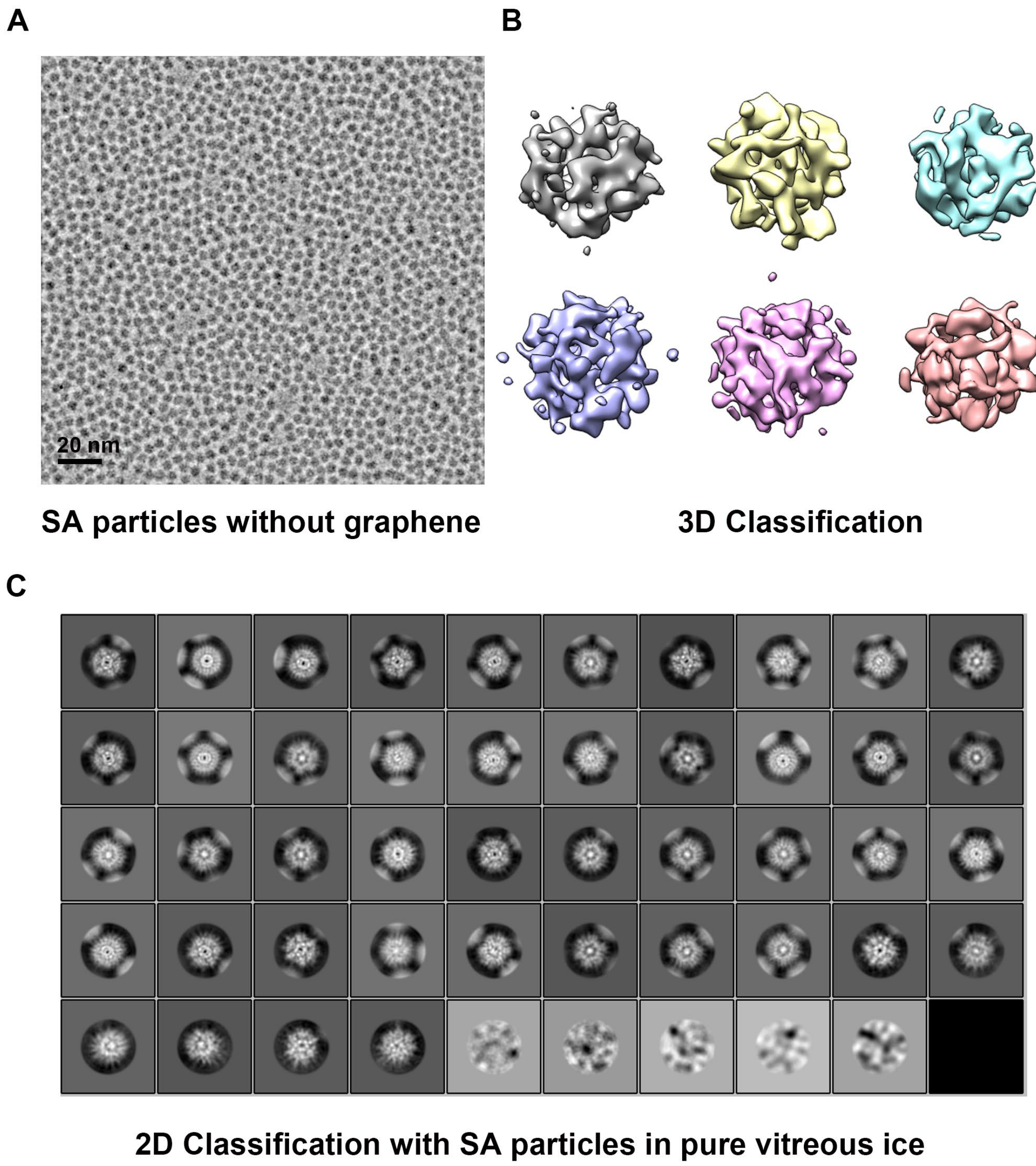

Supplementary Figure 10. Data collection and processing of apo-SA particles in a regular holy carbon grid.

(A) A representative micrograph of collected 1134 apo-SA micrographs in a regular holy carbon R0.6/1 grid by VPP. (B) 3D classification of selected 314,757 particles after 2D classification in (C). No class demonstrates the right SA structure. (C) 2D classification of 1,307,960 auto-picked particles. Class averages indicate a severe preferential orientation.

Supplementary Table 1. Cryo-EM data collection, refinement, and validation statistics

	Apo-state streptavidin	Biotin-bound streptavidin
EMDB ID	EMD-0690	EMD-0689
EMPIAR ID	EMPIAR-10269	EMPIAR-10270
PDB ID	6J6K	6J6J
**Data collection and processing**		
Microscope	FEI Titan Krios	FEI Titan Krios
Detector	Gatan K2 Summit	Gatan K2 Summit
Magnification	215,000x	215,000x
Pixel size (Å)	0.26325	0.26325
Total electron dose (e^{-}·Å^{-2})	50	50
Dose rate (e^{-}·pixel^{-1}·sec^{-1})	6.2	6.2
Defocus range (μm)	-0.8	-0.8
Micrographs collected	1,450	3,309
Micrographs used	1,385	3,272
**Reconstruction**		
Software	Relion	Relion
Total extraced particles	709,967	1,346,980
Number of refined particles	79,289	216,066
Number of final particles	23,991	45,686
Symmetry	D2	D2
Resolution (Å) - FSC 0.5 (unmasked/masked)	4.2/3.8	3.8/3.4
Resolution (Å) - FSC 0.143 (unmasked/masked)	3.8/3.3	3.5/3.2
Map sharpening B-factor (Å^{2})	-100	-70
Local resolution range (Å)	3.0-4.5	2.6-3.8
**Model composition**		
Number of protein residues (atoms)	476 (3548)	476 (3548)
Number of ligand (atoms)	0	4 (64)
**Refinement**		
Software	PHENIX	PHENIX
MapCC (mask/box/peaks/volume)	0.81/0.75/0.74/0.79	0.81/0.77/0.73/0.81
MapCC (ligand)	N/A	0.71
R.M.S deviations		
Bonds lengths (Å)	0.005	0.006
Bonds angles (°)	1.217	1.119
**Validation**		
MolProbity overall score	1.87	1.93
All-atom clashscore	5.37	6.41

Rotamer outliers (%)	1.16	0.00
Ramachandran plot statistics		
Preferred (%)	90.6	88.89
Allowed (%)	6.84	11.11
Outlier (%)	2.56	0.00
EMRinger score	5.62	4.58

Supplementary Table 2. Particles distribution statistics in different processing states.

**Image processing state**	**Total particle numbers in selected 749 micrographs**	**Particle numbers in Subset A (ratio)**	**Particle numbers in Subset B (ratio)**
After 2D Classfication	212,105	134,606 (63.5%)	77,499 (36.5%)
After 3D Classfication	44,326	35,676 (80.5%)	8,650 (19.5%)
Final refinement	13185	10,334 (78.4%)	2,851 (21.6%)
