# Supplementary material for: Single particle cryo-EM reconstruction of 52 kDa streptavidin at 3.2 Angstrom resolution
Source: Nat Commun. 2019 Jun 3;10:2386. doi: 10.1038/s41467-019-10368-w (PMC6546690; doi:10.1038/s41467-019-10368-w)
Supplement: Supplementary file 6 — Description of Additional Supplementary Files [file 41467_2019_10368_MOESM6_ESM.pdf]

Title: Supplementary Movie 1.
Description: Fitting of atomic models in the cryo-EM densities of apo-SA and biotin-SA reconstructions This movie demonstrates the quality of the reconstructed biotin-SA map in different areas with atomic model fit in. It also displays the major difference between biotinSA (white) and apo-SA (green) reconstructions at the biotin binding pocket.

Title: Supplementary Movie 2
Description: Electron tomographic reconstruction of the apo-SA specimen in an area with thick ice This movie is related to Figure 6A-B, Supplementary Figure 7A and 7C, indicating the reconstruction of a ~50 nm thickness vitreous apo-SA sample on graphene grid. The reconstruction clearly shows the air-water interface (AWI), graphenewater interface (GWI) and the interlayer vitreous ice with different protein distribution behavior.

Title: Supplementary Movie 3
Description: Electron tomographic reconstruction of the apo-SA specimen in an area with thin ice This movie is related to Supplementary Figure 7B and 7D, indicating the reconstruction of a ~10 nm thickness vitreous apo-SA sample on graphene grid. The protein distribution in the area with ~10 nm thickness is similar to the ~50 nm area (Supplementary Movie 2), but with a thinner interlayer vitreous ice.
